# Antimony Toxicity

**DOI:** 10.3390/ijerph7124267

**Published:** 2010-12-20

**Authors:** Shyam Sundar, Jaya Chakravarty

**Affiliations:** Department of Medicine, Institute of Medical Sciences, Banaras Hindu University, Varanasi 221 005, India; E-Mail: tapadar@gmail.com

**Keywords:** antimony, toxicity, leishmaniasis, occupational hazard

## Abstract

Antimony toxicity occurs either due to occupational exposure or during therapy. Occupational exposure may cause respiratory irritation, pneumoconiosis, antimony spots on the skin and gastrointestinal symptoms. In addition antimony trioxide is possibly carcinogenic to humans. Improvements in working conditions have remarkably decreased the incidence of antimony toxicity in the workplace. As a therapeutic, antimony has been mostly used for the treatment of leishmaniasis and schistosomiasis. The major toxic side-effects of antimonials as a result of therapy are cardiotoxicity (~9% of patients) and pancreatitis, which is seen commonly in HIV and visceral leishmaniasis co-infections. Quality control of each batch of drugs produced and regular monitoring for toxicity is required when antimonials are used therapeutically.

## 1. Introduction

Antimony (symbol Sb from the latin *stibium*) is a silvery white metal with atomic number 51, that is found in the earth’s crust. Its main applications are industrial. Elemental antimony can be used for producing semiconductors, infrared detectors and diodes. Because of its relative inflexibility, it is usually mixed into alloys for further application, e.g., manufacture of lead storage batteries, solder, sheet and pipe metal, bearings, castings and pewter, *etc.* Antimony oxide can be used in fire-retardant formulations for plastics, rubbers, textiles, paper and paints whereas antimony trisulfide is used in the production of explosives, pigments, antimony salts and ruby glass [1–3]. Antimony compounds have been used as medicines since their introduction by the alchemist John of Rupescissa in the 14th century [4], mainly in the treatment of two parasitic diseases, leishmaniasis and schistosomiasis.

Antimony and its compounds are naturally present in the Earth’s crust and are released into the environment by natural discharges such as windblown dust, volcanic eruptions, sea spray, forest fires, and biogenic sources. The concentration of antimony in air ranges from a nanogram per cubic meter (ng/m^3^) to about 170 ng/m^3^. The concentration of antimony that is found dissolved in rivers and lakes is usually less than 5 parts of antimony in 1 billion parts of water (ppb) and it is found attached to particles of dirt. Antimony occurs predominantly in the pentavalent state in aerobic fresh water and sea water and the trivalent state is more common under anaerobic conditions as well as a results of anthropogenic activities. A U.S. geological survey showed that soil concentrations range from less than 1 to 8.8 ppm, with a mean of 0.48 ppm [5]. The average intake of antimony from food and water was estimated to be roughly 5 μg/day in a study [6]. These data show that the general population is exposed to low levels of antimony. Toxicity may arise during occupational exposure, domestic use or when it is used as a therapy. This review discusses antimony toxicity arising from occupational exposure and when it is used as a therapy.

## 2. Results and Discussion

### 2.1. Antimony as an Occupational Hazard

Occupational exposure to antimony occurs mainly in workers involved in industries producing antimony and antimony trioxide, metal mining, smelting and refining, coal-fired power plants, refuse incineration, or those working in indoor firing ranges. Most of the data of antimony toxicity comes from the time when primitive work conditions prevailed and there was no adequate protection for the workers. Another problem in assessing its toxicity industrially is that arsenic and lead are often found with it, and other toxic materials may also be produced in the course of the process, and separation of exposures may be difficult or impossible.

#### 2.1.1. Inhalational Exposure

Health effects have been observed following inhalational exposure to several antimony compounds e.g., antimony trioxide, stibine (antimony hydride), antimony trisulfide, antimony pentoxide, antimony trichloride, antimony pentasulfide, metallic antimony, *etc.* The absorption of antimony from the respiratory tract is a function of particle size. Aerosols containing small particles composed of antimony compounds with low water solubility (e.g., particles of antimony oxides) are retained in the lungs for a longer period of time than those containing larger particles with high water solubility (e.g., particles of antimony tartrate) [7,8].

##### 2.1.1.1. Respiratory Effects

Chronic exposure to antimony trioxide and/or pentoxide dust (8.87 mg antimony/m^3^ or greater) was seen to cause pneumoconiosis, however, these workers were also exposed to a variety of other compounds like arsenic oxide, iron oxide, hydrogen sulfide, and sodium hydroxide [9,10]. Antimony pneumoconiosis was also described by Karajovic in a population of antimony miners and smelters in Yugoslavia based on diffuse x-ray opacities but this was also confounded by simultaneous silicosis [11]. Other respiratory effects reported in workers include chronic bronchitis, chronic emphysema, inactive tuberculosis, pleural adhesions, and respiratory irritation (characterized by chronic coughing, wheezing and upper airway inflammation) [10]. Similar findings were seen in animal studies [12].

##### 2.1.1.2. Cardiovascular Effects

Cardiovascular effects in humans are supported by the finding of cardiac effects following parenteral administration of antimony to humans. Increased blood pressure and altered ECG (electrocardiography) readings mostly of the T-waves were observed in workers exposed to 2.15 mg antimony/m^3^ as antimony trisulfide for 8 months to 2 years; however these workers had also been exposed to phenol formaldehyde resin. Inhalation exposure to antimony trisulfide dust was also seen to result in degenerative changes in the myocardium and related ECG abnormalities in variety of animal species [13]. Nevertheless, evidence of heart disease from industrial exposure to antimony is not very strong.

##### 2.1.1.3. Gastrointestinal Effects

Repeated prolonged exposure to airborne antimony trichloride [14], antimony trisulfide [13] or antimony oxide [15] was seen to cause abdominal pain, diarrhea, vomiting, and ulcers. A causal relationship to antimony exposure has not been definitely established because workers were exposed to a variety of other agents in addition to antimony that might cause or contribute to gastrointestinal effects (e.g., hydrogen chloride, sodium hydroxide).

##### 2.1.1.4. Dermal effects

Airborne antimony has effects in skin described as “antimony spots” which are pustules and eruptions in the trunk and limbs near sweat and sebaceous glands. This dermatitis is more commonly seen in association with hot weather and in workers exposed to high temperatures [10,16,17]. Transferring the worker to a cooler environment often resulted in the rash clearing up within 3–14 days.

##### 2.1.1.5. Reproductive Effects

Two thirds of rats exposed to 209 mg antimony/m^3^ as antimony trioxide for 63 days failed to conceive. An increased incidence of spontaneous abortions and disturbances in menstruation, were reported in women working at an antimony metallurgical plant as compared to a control group. The women were exposed to a mixture of antimony trioxide, antimony pentasulfide, and metallic antimony [18].

##### 2.1.1.6. Carcinogenicity

There is inadequate evidence for carcinogenicity of antimony trioxide and trisulphide in humans but antimony trioxide and antimony trisulfide have been seen to cause lung tumours in rats. Antimony trioxide is classified as possibly carcinogenic to humans (Group 2B) by the International Agency for Research on Cancer [3].

##### 2.1.1.7. Genotoxicity

The results of *in vitro* genotoxicity studies have shown positive results for chromosome breakage in human leukocytes [19]. In a study to assess genotoxic risk and oxidative damage in workers exposed to antimony trioxide, there was no difference in sister chromatid exchange and micronuclei between those exposed and controls however, increased oxidative damage to DNA was observed in the exposed group [20]. In a recent study, antimony showed genotoxicity in both bacterial mutation tests and chromosomal aberration tests in cultured mammalian cells [21]. Due to lack of *in vivo* studies genotoxicity in humans cannot be determined at this time.

#### 2.1.2. Oral Exposure

Historically, antimony has been known for its emetic properties. Amounts as low as 0.529 mg/kg can result in vomiting. Oral exposure to antimony predominantly affects the gastrointestinal system. Seventy people became acutely ill after drinking lemonade containing 0.013% antimony. The lemonade had been prepared and left overnight in buckets coated with enamel containing 2.88% antimony trioxide. Fifty-six people were taken to the hospital with burning stomach pains, colic, nausea and vomiting. Most recovered within 3 hours, but in some cases recovery was not complete for several days [22]. It is estimated that a person consuming 300 mL of lemonade would have received a dose of approximately 36 mg antimony, or approximately 0.5 mg/kg for a 70-kg adult. No other toxicity has been recorded in humans for oral exposure to antimony.

#### 2.1.3. Other Exposure from Antimony Compounds

Antimony was implicated in the cause of cot deaths, or Sudden Infant Death Syndrome (SIDS) by Richardson in 1990 [23]. It was claimed that antimony compounds used in fireproofing cot furnishings amongst other additives was primarily responsible for SIDS due to the action of a fungus (*Scopulariopsis brevicaulis*) growing on polyvinyl chloride cot mattress covers. *In vitro* experiments appeared to demonstrate the release of stibine and phosphine, hydrides of antimony oxide fire retardant and phosphorus plasticisers from polyvinyl chloride mattress covers which had been treated with these substances and it was claimed that they had caused deaths from their toxicity. However, urine antimony concentration of antimony in infants dying from SIDS were similar to values found in control infants and healthy infants [24]. This causal role of antimony in SIDS was ultimately refuted due to lack of evidence.

Recently the National Institute for Occupational Safety and Health investigated a possible outbreak of antimony toxicity wherein 30 firefighters reported elevated antimony levels on hair analyses as some fire fighter station uniforms contain the flame-retardant antimony trioxide. However, no differences in urine antimony levels between departments wearing and not wearing this uniform were detected. It was hence concluded that wearing antimony-containing uniforms does not pose a risk for antimony toxicity [25]. The CDC stated in their report that only validated methods should be used for the determination of antimony toxicity. Urine testing is the most accurate, reliable, and valid test method for measuring antimony levels in the body. CDC has established ranges for urine levels of antimony in the U.S. population: 0.120–0.364 micrograms/gram creatinine. Hair testing is not a validated method for heavy metals testing (which includes antimony), and is not recommended [26].

#### 2.1.4. Prevention of Exposure to Antimony

To safeguard the general public in USA, the Environmental Protection Agency (EPA) allows 0.006 parts of antimony per million parts of drinking water [27]. In the guidelines for Drinking-water Quality, WHO established a tolerable daily intake (TDI) of 6 μg/kg bodyweight/day for antimony [28].

The EPA requires that discharges or spills into the environment of 5,000 pounds or more of antimony be reported. The Occupational Safety and Health Administration (OSHA) has set an occupational exposure limit of 0.5 milligrams of antimony per cubic meter of air (0.5 mg/m^3^) for an 8-hour workday, 40-hour workweek [5].

### 2.2. Antimony in Therapeutics

Antimony had a reputation of being a universal panacea of all kinds of diseases in the middle ages. In 1631, the German alchemist Adrian Von Mynsicht successfully described potassium antimony tartrate [29]. Since the last century antimonials have been used for the treatment of two parasitic diseases schistosomiasis and leishmaniasis.

#### 2.2.1. Schistosomiasis

A number of antimony compounds have been used for the treatment of schistosomiasis e.g., sodium antimony tartrate, sodium antimony dimercaptosuccinate (stibocaptate, Astiban) sodium antimonyl gluconate, *etc.* [30,31]. In a comparative trial of three antimonials used in schistosomiasis, 78% patients had gastrointestinal symptoms e.g., vomiting, anorexia. Arthralgia was common in all the three groups, but severity of the symptoms were more in patients taking antimony sodium tartrate (AST) and sodium antimonyl gluconate (TSAG). Complete flattening or frank inversion of T waves in ECG was seen in 68%, 67% and 42% of TSAG, AST and TWSb (antimony dimercaptosuccinate) respectively. Although substernal pain was common only three cases on i.v. sodium antimony tartrate had an acute vascular collapse. Cough was a common symptom however pneumonia occurred in two patients and hepatotoxicity was observed in 1.9% patients [31]. With the advent of the more efficacious and less toxic alternative praziquantel, the trivalent antimonials were phased out from the treatment of schistosomiasis in the 1970s.

#### 2.2.2. Leishmaniasis

In 1912, Vianna successfully used tartar emetic to cure cutaneous leishmaniasis for the first time [32]. In 1915 it was used for visceral leishmaniasis for the first time. Tartar emetic, although extremely effective in the treatment of leishmaniasis, was abandoned due to its toxicity. Injections led to severe vomiting and retching, apart from that pneumonia, joint pain, lung, kidney and bowel complication was also reported [33]. In 1923 the first pentavalent antimony compound urea stibamine was discovered which had much less toxicity than its trivalent predecessor. Later it was replaced by sodium stibogluconate which to date remains the drug of choice for the treatment of visceral leishmaniasis worldwide, except in North Bihar where a high level of antimony resistance exists.

Treatment with sodium antimony gluconate at the therapeutic dose may result in minor side effects such as arthralgia, myalgia, transient elevation of hepatocellular enzyme levels, and minor ECG changes [34,35]. A decrease in the height of T waves and T-wave inversion is seen in about 50% of patients [35]. Although serious cardiotoxicity is uncommon, occurring in less than 9% cases, death may result in these patients [34,36–38]. Features of dangerous cardiotoxicity include a concave ST segment and prolongation of the corrected QT interval (QTc), which is measured by dividing the QT interval by the square root of the preceding interval between the preceding two QRS complexes (RR interval) [39]. Normal QTc values are less than 0.37 s and 0.44 s for males and females, respectively, while an increase of 0.03 s or an absolute value of greater than 0.50 s are considered ominous [34,35].

In a cluster of cases with cardiotoxicity due to sodium antimony gluconate, the first dangerous sign of cardiotoxicity was prolonged QTc, followed by multiple ventricular ectopics, then ventricular tachycardia, torsade de pointes, ventricular fibrillation, and potentially death. In this series cardiotoxicity occurred at a much lower cumulative dose than previously reported and was associated with higher osmolarity of the batch of drugs. Elevated osmolarity reflects an increased concentration of particles, and thus reflect both the concentration of the drug and its state of aggregation. However, in this study implicated lot had comparable antimony contents as the effective lot, implying some other cause for the increase in their osmolarity. Incorrect formulation e.g., incorrect antimony to gluconate ratio and the presence of trivalent antimony in a preparation could also result in increased osmolarity, This study suggests that higher than expected osmolarity may serve as a nonspecific indicator of a problem in formulation [40]. Another outbreak of fatal cardiotoxicity occurred in Nepal amongst visceral leishmaniasis patients treated with a recently introduced batch of generic sodium stibogluconate. Eight (36%) of 23 patients treated with this batch died, and five deaths (23%) were attributed to the cardiotoxicity of the drug [41].

The exact mechanism for cardiotoxicity is not known, but antimonials prolong the action potential of guinea pig ventricular myocytes at therapeutically relevant concentrations for the treatment of leishmaniasis via an increase in cardiac calcium currents. In the heart, calcium currents regulate the plateau phase of the cardiac action potential and increased amplitudes produce a delay in cardiac repolarization, which may explain the propensity of patients treated with antimonial compounds to develop QT prolongation and life-threatening arrhythmias [42]. It is now generally accepted that all pentavalent antimonials are prodrugs that require biological reduction to the trivalent form [Sb(III)] for antileishmanial activity. The site (amastigote or macrophage) and mechanism of reduction (enzymatic or nonenzymatic) remain controversial [43]. Although the exact molecular mechanism(s) underlying the increase in calcium currents observed with trivalent antimony is not known, it is postulated that high affinity for sulfhydryl groups may affect calcium channels by oxidizing cysteine residues located either directly on the channel protein or on a closely associated protein [44].

Pancreatitis is another adverse effect of pentavalent antimonials. Incidence of all adverse events and especially of pancreatitis is quite high in HIV and VL coinfection. In a study of 25 HIV-VL coinfected patients, adverse effects were observed in 56%. In seven (28%), treatment with meglumine antimoniate (MA) was discontinued permanently due to serious adverse effects that included acute pancreatitis (5), acute renal failure (1), and leukopenia (1). Three (12%) patients died during therapy due to severe acute pancreatitis attributable to MA. Patients who developed acute pancreatitis were not alcohol drinkers, their triglyceride levels were not elevated and the biliary tree was normal on abdominal ultrasound examination [45]. The reasons behind increased pancreatitis in these patients could be multiple. Amount of antimony has been seen to vary with different batches which could lead to use of higher doses of Sb^V^ than intended [46]. Subclinical pancreatitis frequently occurs in patients with AIDS which could predispose them to pancreatic disease [47,48]. Elevation of pancreatic and liver enzymes were also observed in a study of new world cutaneous leishmaniasis in which pentavaalent antimonials were given at the dose of 20 mg/kg/day for 20 days. The most prominent complaints, myalgia and abdominal pain, were reported by 29–56% and 4–25% of the patients, respectively. Another 46–69% had mild-to-moderate elevations and 2–19% had grade 3 elevations of pancreatic enzymes, whereas, 44–75% had mild-to-moderate elevations and 4–12% had grade 3 elevations of liver enzymes [49].

In Brazil higher frequency of skin reactions was observed in some patients of cutaneous leishmaniasis treated with meglumine antimoniate. This lot of drugs had lower pH and osmolarity and higher concentrations of total and trivalent antimony, lead, cadmium and arsenic and the skin reaction was attributed to the heavy metal contamination [50].

## 3. Conclusions

With dramatic improvements in working conditions in antimony processing and stringent guidelines antimony has largely ceased to be a common occupational health hazard, however, constant vigilance is required for emerging toxicity.

As for its use as a therapeutic agent, continuous quality control of each batch of drugs produced should be done to ensure safety. The simple technique of measuring of osmolarity may help identify inappropriately manufactured drugs. Regular monitoring for toxicity needs to be done. Use of alternate less toxic drugs for the treatment of leishmaniasis especially in HIV co-infected patients could be another strategy. Availability of drugs like miltefosine, paramomycin and liposomal Amphotericin B (L-AmB) makes this a viable option. Moreover, a preferential pricing agreement with WHO (agreement between Gilead and WHO of 14 March 2007) has recently reduced the price of L-AmB (AmBisome®) for endemic regions to $20 per 50-mg vial [51]. This preferential pricing further opens the prospect of either single dose treatment or short course combination regimens. Combining multiple drugs not only reduces toxicity of individual drugs but also reduces drug pressure, decreases duration of treatment leading to better compliance, decreased hospital stay and ultimately cost of therapy. Paromomycin was one of the first drug to be used in combination with pentavalent antimony compounds in the 1990s in Kenya, India and Sudan [52–54]. This combination is currently in use in Sudan [55]. Well conducted trials of combination therapy are the need of the hour to increase efficacy, decrease toxicity and prevent resistance.
